# Effects of follicle-stimulating hormone on fat metabolism and cognitive impairment in women during menopause

**DOI:** 10.3389/fphys.2022.1043237

**Published:** 2022-12-05

**Authors:** Liwei Mao, Lian Wang, Samuel Bennett, Jiake Xu, Jun Zou

**Affiliations:** ^1^ School of Kinesiology, Shanghai University of Sport, Shanghai, China; ^2^ School of Biomedical Sciences, The University of Western Australia, Perth, WA, Australia

**Keywords:** follicle-stimulating hormone, adipogenesis, cognitive impairment, Alzheimer’s disease, menopausal women

## Abstract

Lipid metabolism disorder is a common pathological manifestation of menopausal women, and is also an important risk factor for many diseases at this stage of life. Epidemiological studies have shown that high levels of follicle-stimulating hormone (FSH) in menopausal women are closely associated with changes in body composition, central obesity, and cognitive decline. Exogenous FSH causes growth and proliferation of adipose, whereas blockage of the FSH signaling pathway leads to decline in adipose. Mechanistically, FSH, FSH receptor (FSHR), G protein coupling, gene mutation and other pathways are involved in adipogenesis and cognitive impairment. Here, we review the critical role and potential interactions of FSH in adipogenesis and cognitive impairment in menopausal women. Further understanding of the exact mechanisms of FSH aggravating obesity and cognitive impairment may provide a new perspective for promoting healthy aging in menopausal women.

## 1 Introduction

Stages of Reproductive Aging Workshop (STRAW) defined menopausal transition (MT) as the onset of menstrual cycle fluctuations (more than 7 days above normal) accompanied by a continuous follicle-stimulating hormone (FSH) rise until the end of the last menstrual period (Practice Committee of the American Society for Reproductive Medicine, 2006). The period from the beginning of MT to 1 year after the end of the final menstrual cycle is called perimenopause. There is some variation in the age at which MT begins, generally between 44 and 55 years of age worldwide, due to different ethnic regions and increased life expectancy for women (Takahashi et al., 2015). The normal MT lasts about 3–9 years, while postmenopausal phase will account for 30–40% of life (Zhu et al., 2018). The cluster of physical and psychological symptoms that appear in women during MT and early postmenopausal period is called climacteric syndrome, including paramenia, vasomotor symptoms (hot flashes, night sweats), autonomic dysfunctions (dizziness, headache, and insomnia), and neuropsychiatric symptoms (anxiety, depression, and memory loss) (Avis et al., 1982; Brenner, 1988; He et al., 2021a).

**FIGURE 1:**
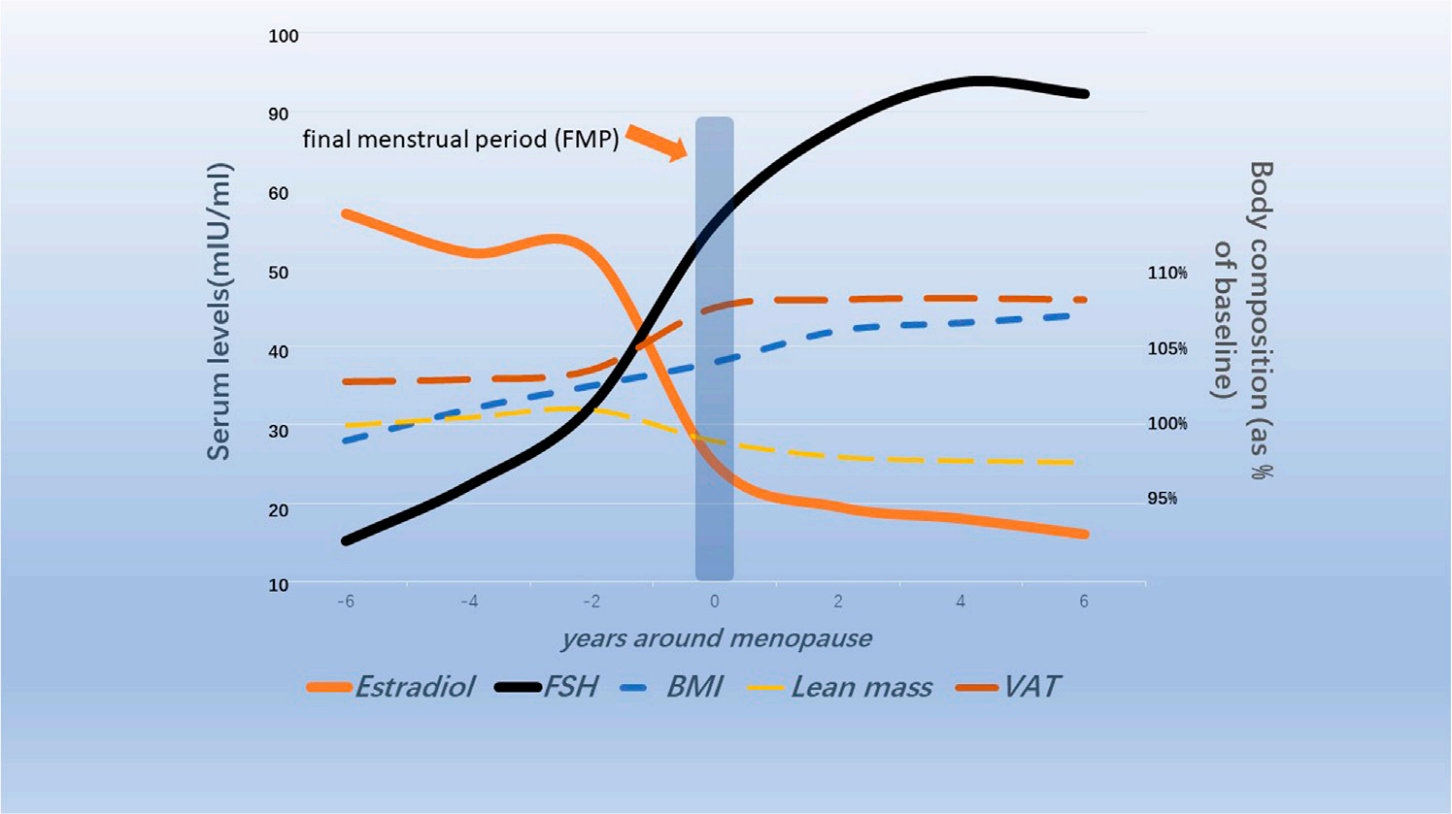
Schematic diagram of changes in ovarian hormones and body composition in menopausal transitional women (Randolph et al., 2011; Greendale et al., 2019b; Greendale et al., 2021).

Hormone disorder is an important cause of climacteric syndrome. Estrogen levels continued to decline and FSH levels continued to rise during menopause and early postmenopause, according to a 3-year observational study of 3,257 perimenopausal women conducted by the Women’s Health Across the Nation (SWAN) (Randolph et al., 2004). Further observation showed that FSH began to increase 6 years before menopause, rapidly increased 2 years before the final menstrual period, and stabilized 2 years after menopause. Estrogen drops sharply in the 2 years before menopause (Randolph et al., 2011) (Figure 1). Changes in estrogen and FSH concentrations during perimenopause are influenced by ethnic differences and body mass index (BMI). The study found that non-Hispanic white postmenopausal women had higher baseline levels of bioavailable estrogen than Hispanic and African American women. However, the decline in estrogen levels was more pronounced in non-Hispanic white women without hormone therapy after menopause (Kim et al., 2012). Studies have found that central obesity is closely related to hormone level disorders, including sex hormones, growth hormones, epinephrine, thyroid hormones and so on (Björntorp, 1995; Ziegler et al., 2012). A longitudinal study of SWAN followed 1,246 racially diverse participants with an average age of 47.1 years over time. It was demonstrated that fat mass and BMI began to increase from 5 to 6 years before menopause to 4 years or more after menopause, while lean mass proportion continued to decrease during this period (Greendale et al., 2019a) (Figure 1). MT is associated with the occurrence of central adiposity, and there are ethnic differences. There is a difference in the proportion of body fat in white women compared with black women and Japanese women before and after menopause (Greendale et al., 2021). Abnormalities in energy and lipid metabolism are the predisposing factors of weight gain in menopausal women (Ko and Jung, 2021). Therefore, a variety of hormone replacement therapies were developed to prevent and ameliorate lipid and energy metabolic disorders in postmenopausal women, but the effects are not entirely consistent (Yüksel et al., 2006; Odabasi et al., 2007; Li et al., 2021a).

In addition, cognitive function of is also affected by menopause, which increases the susceptibility of menopausal women to Alzheimer’s disease (AD) (Christensen and Pike, 2015). Lisa et al. used positron emission tomography (PET) and structural MRI to monitor brain structure and metabolic changes in women aged 40–65. Subjects were divided into pre-menopausal (average age 44), peri-menopausal (average age 50) and post-menopausal group (average age 57). It found that brain energy metabolic load increased from MT to postmenopausal period, which was manifested by increased ATP production and blood flow. However, brain connectivity, white mass, and glucose metabolism reduced significantly, and Amyloid-β (Aβ) deposition was aggravated (Mosconi et al., 2021). A 3-year longitudinal study based on PET and structural MRI found that women showed metabolically degenerative AD development and deterioration during MT (Mosconi et al., 2018). To be clear, menopausal lipid dysfunction and cognitive impairment are not independent of each other. Estrogen deficiency plays a leading role in obesity and cognitive deterioration in menopausal women (Lizcano and Guzmán, 2014; Leeners et al., 2017; de Lange et al., 2020). Estrogen related receptor alpha promotes adipogenic genes expression during adipocyte differentiation (Ijichi et al., 2007). Estrogen directly upregulates α2A adrenergic receptor in human adipose tissue through estrogen receptor alpha, which controls lipolysis and affects fat distribution in women (Pedersen et al., 2004). A study showed that a new type of estrogen receptor β is expressed in different adipose tissues (Pedersen et al., 2001). However, there are differences in the expression of estrogen β receptor subtypes in human adipose tissue. The expression of β-1 receptor in intra-abdominal adipose tissue was significantly reduced compared with subcutaneous adipose tissue. A large number of studies showed that high-fat diet-induced neuroinflammation, oxidative stress, and mitochondrial damage in nerve cells significantly impair brain cognitive function (Wang et al., 2018; Tan and Norhaizan, 2019; Maneechote et al., 2022). A study showed that middle-aged female rhesus monkeys on a high-fat diet after ovariectomy suffer significant brain damage, while estradiol supplementation protects against neuroinflammation, Aβ deposition and tau tangles (Cervera-Juanes et al., 2022). Notably, in addition to estrogen, recent studies have shown that FSH is associated with fat metabolism disorders and cognitive impairment during MT, making it a promising target for the treatment of menopausal obesity and AD.

## 2 Biogenesis and gonadal role of FSH

**FIGURE 2:**
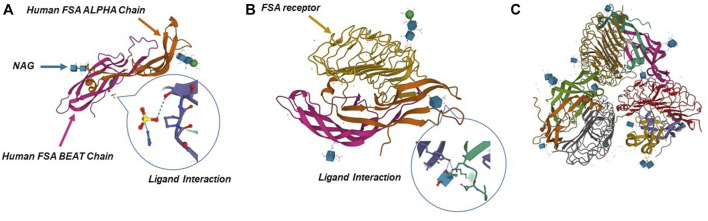
Crystal structure of human FSH complexed with its receptor. **(A)** 3D structure of human FSH. **(B,C)** Structure of FSH in complex with the entire ectodomain of FSHR. Pictures refer to Protein Data Bank database (1FL7, 1XWD, and 4AY9).

FSH, luteinizing hormone (LH) and thyroid stimulating hormone (TSH) are three glycoprotein hormones synthesized and secreted by the anterior pituitary gland. FSH and LH are also collectively known as gonadotrophins (Chappel et al., 1983). Their synthesis and secretion are in response to hypothalamic gonadotropin-releasing hormone (GnRH) (Chappel et al., 1983; Haldar et al., 2022). FSH is a complex heterodimeric protein with a molecular weight of 35.5 kDa, consisting of a functional common α-subunit (92–96 amino acids) and a hormone-specific FSH β-subunit (110–118 amino acids) with non-covalent bonds (Ulloa-Aguirre and Timossi, 1998; Ulloa-Aguirre et al., 1999). The FSH receptor (FSHR) gene is located on chromosome 2 with 52 kbp length and FSHR protein is a G protein-coupled receptor (Figure 2). FSHβ ensures functional specificity of FSH by recognizing and binding different FSHR (Jiang et al., 2012). FSHR consists of four different isoforms (FSHR1- FSHR4) according to the different alternate splicing sites of the FSHR gene, among which FSHR1 and FSHR3 are active in multiple functional activities of FSH (Bhartiya and Singh, 2015; Bhartiya et al., 2021).

Clinical FSH preparations were first extracted from the pituitary gland in the 1960s, and human menopausal gonadotropin containing FSH and LH extracted from postmenopausal urine became the standard preparation for the next 30 years (Loumaye et al., 1995). By the 1990s, FSH was prepared *in vitro* using recombinant DNA technology and its biological activity was regulated by epigenetic modification of the common α-subunit and FSHβ (Fauser, 1998; Bousfield and Harvey, 2019). The recombinant FSH was completely stripped of LH, with better specific activity and purity, ensuring batch consistency.

GnRH pulse release stimulates FSH secretion, and inhibin B and estradiol are the main inhibitory hormones of FSH secretion (Grunewald et al., 2013; Yding Andersen, 2017). FSH induces follicle growth and coregulates the proliferation and maturation of germ cells with LH (Howles, 2000; Palermo, 2007; El-Hayek et al., 2014). An animal study found that FSH promotes the production of estradiol required for reproduction but does not stimulate prepubertal follicle overgrowth (François et al., 2017). In addition, FSH was found to promote synthesis and secretion of progesterone in granulosa cells, which may affect the prognosis of *in vitro* fertilization (Oktem et al., 2017). In male, FSH and testosterone act through Sertoli cells, and FSH is essential for inducing and maintaining normal sperm production. Meanwhile, FSH regulates mitosis and proliferation of Sertoli cells and is essential for the determination of Sertoli cells (Simoni et al., 1999).

The gonadal role of FSH in women of growing and reproductive age is well established. However, recent studies have found that high levels of FSH in menopausal women are closely related to lipid metabolism disorders and cognitive impairment.

## 3 Epidemiological characteristics of FSH level and body composition change in menopausal women

MT is associated with changes in body composition and central obesity. Subcutaneous and visceral fat are the main stores of white fat in the body. Visceral adipose tissue (VAT), as an endocrine organ, produces proinflammatory adipose cytokines, exacerbating metabolic disturbance typical of menopause. A study showed a positive correlation between epicardial fat and visceral fat, BMI, waist circumference in menopausal women (Fernández Muñoz et al., 2014). Compared with premenopausal women, menopausal women show a higher prevalence of metabolic syndrome, which is closely associated with obesity, inadequate life habits and vasomotor symptoms (Blanco et al., 2020; Li et al., 2021b). The study found that overweight menopausal women had higher serum levels of insulin, C-peptide, resistin, leptin and inflammatory factors than normal-weight menopausal women (Palla et al., 2020). In addition, FSH levels were found to be significantly associated with adiponectin and leptin-adiponectin ratio in menopausal women, which may link FSH to abnormal energy metabolism and insulin resistance (Huang et al., 2020). Cohort studies based on Asian women found that obesity is closely related to the incidence and severity of psychological and physical climacteric symptoms (Lin et al., 2005; Kim et al., 2020). Estrogen has important protective effect on adipose tissue distribution and adipose metabolism. As estrogen levels drop after menopause, women begin to accumulate visceral fat rapidly, forming central obesity (Bjune et al., 2022). Since the decrease of estrogen and the increase of FSH level in MT have a high coincidence period, and plasma estrogen level and ovarian hormones are important factors affecting FSH. Therefore, further high-quality clinical studies are needed to determine whether FSH plays a primary role or a supporting role in this process.

An earlier study (84 normal-weight and 46 severely obese women) found that FSH concentrations in obese women began to increase 4 years earlier during the MT than in normal-weight women, accompanied by premature ovarian failure (Klinga et al., 1982; Klinga et al., 1983). Margaret et al. found that FSH was independently associated with low lean body mass in 94 menopausal women aged 50–64 years (*β* = −0.099) after correction for age, race, menopause duration, bioavailable estradiol, testosterone, LH and many other factors (Gourlay et al., 2012). In older menopausal women (238 women, mean age 81 years), subjects with higher FSH levels showed lower body weight (−8.4%), lean mass (−6.1%), total fat mass (−11.9%), VAT (−17.6%), spinal BMD (−8.6%) and increased bone marrow adiposity (+8.4%) (Veldhuis-Vlug et al., 2021). A Chinese study found circulating FSH levels in menopausal women were about ten times higher than in premenopausal women, and FSH levels in males older over 60 were threefold higher than in males under 45. Further observation found that BMI was associated with circulating FSH levels in 413 men aged 61 to 65 and 499 women aged 51 to 55, especially in women (Liu et al., 2015).

In a longitudinal study (667 menopausal women), FSH increased by a maximum of 33.9 mIU/ml in menopausal women who discontinued hormone therapy, and elevated FSH was associated with increases in body fat, body fat mass, and subcutaneous fat percentage (Mattick et al., 2022). However, Wu recently found no correlation between FSH and changes in bone mass and body composition (Wu et al., 2021). The inconsistency of these results reflects the complexity of micro-regulation to macro manifestations. Moving forward, the studies adjusted for factors such as age, race and timing of menopause, but inevitably included estrogen interference, one of the barriers to clinical research. High-quality basic research can help reveal the independent role of FSH in lipid metabolism disorders during the MT.

## 4 Potential mechanisms by which FSH impacts fat metabolism

### 4.1 Pleiotropic signaling ability of FSHR drives FSH to regulate fat metabolism

The internal regulation of FSH on FSHR directly affects the activation and inhibition of downstream pathways. Early *in vivo* and *in vitro* studies showed that addition of FSH to Sertoli cells reduced the expression of FSHR within 8 h, but not in a dose-dependent manner, and FSHR expression was upregulated in hypophysectomized rats (Maguire et al., 1997). However, preadipocytes extracted from chicken adipose tissue formed adipocyte morphology more quickly after exposure to FSH, with significantly upregulation of FSHR expression and activation of peroxisome proliferator-activated receptor gamma (PPARγ) signaling pathway (Cui et al., 2012). Together, the inconsistency between FSH and FSHR may be a self-regulating mechanism to maintain the functional balance of FSH and may be related to cell and tissue specificity.

**FIGURE 3:**
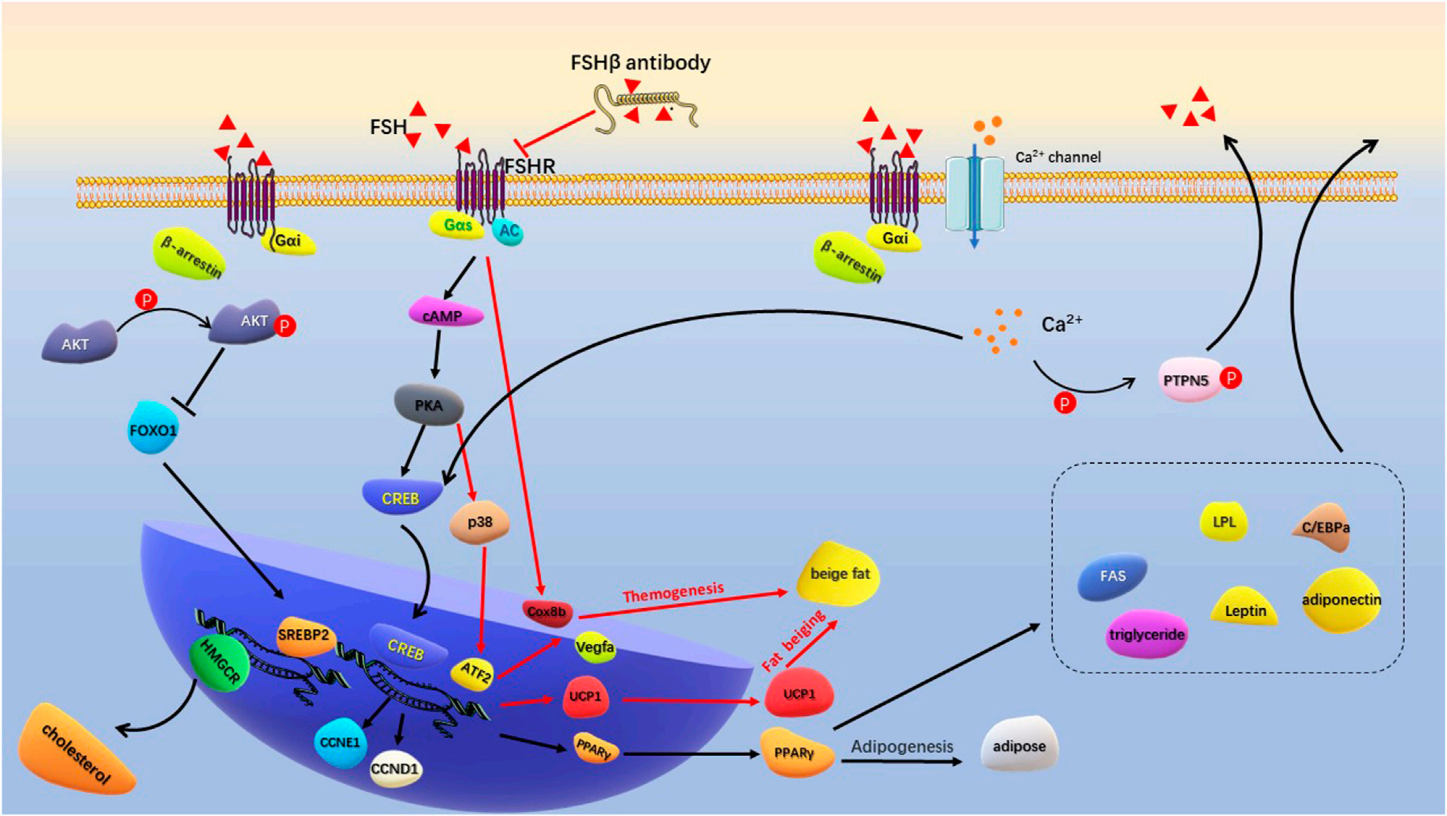
Schematic diagram of FSH regulating adipogenesis. FSH binds to Gas-coupled FSHR to activate the classical cAMP/PKA/CREB pathway. Increased PPARγ transcription promotes production of lipid and a series of lipogenic factors. Specific FSHβ antibodies inhibit FSH-FSHR interactions, activate transcription of Cox8b, Vegfa, and UCP1 to promote beige fat production and thermogenesis. FSH binds to Gαi-coupled FSHR to activate Ca^2+^ channel. Intracellular calcium influx promotes lipid generation by activating the CREB pathway, and assists GnRH in promoting FSH secretion by phosphorylation of PTPN5. The binding of FSH to Gαi-coupled FSHR activates p-Akt in the presence of β-arrestin. By inhibiting FoxO1, it promotes the transcription of SREBP-2 and HMGCR genes to produce cholesterol.

FSH and FSHR are targets for regulating fat metabolism, and the development of antigens targeting FSHβ has become a research hotspot. A short FSHβ amino acid chain was developed to competitively bind to the FSHβ, thereby reducing the interaction between FSH and FSHR (Han et al., 2020). The antigen not only prevented weight gain and fat accumulation in ovariectomized mice, but also significantly reduced fat accumulation in normal female and male mice. Mechanistically, the PPARγ adipogenic signaling pathway was inhibited by FSH antigen injection, and the adipogenic thermogenesis was enhanced by upregulating uncoupling protein 1(UCP1) expression in visceral and subcutaneous tissues. Sakshi et al. recently developed the first humanized FSH blocking antibody that binds to human FSHβ with high specificity (Gera et al., 2020). Cell culture experiments showed that FSH antigen reversed the concentration of FSH and increased the expression of genes associated with beiging (Cox8b and UCP1). FSHβ polyclonal antibody (epitope: LVYKDPARPKIQK) significantly reduced adipose tissue in high-fat diet induced obese mice and ovariectomized mice. This antibody activated UCP1 by blocking the interaction between FSH and FSHR (Liu et al., 2017). *In vivo*, FSH antibody increased UPC1 expression in white adipose tissue (WAT) and brown adipose tissue (BAT), and increased expression of Cidea, C/EBPα and Vegfa genes in BAT was consistent with activation of thermogenic genes. Overexpression of UCP1 and enhanced mitochondrial biogenesis increased adipose tissue thermogenesis (Figure 3). Overall, by competitively binding to FSHβ, FSH antigens effectively inhibited adipogenesis and enhanced adipogenic thermogenesis. The development of safe and effective FHS antigens or high affinity ligands for FSHR is an important direction for the intervention of postmenopausal obesity.

### 4.2 Gαi/Ca^2+^/CREB signaling pathway mediates FSH-induced lipid biosynthesis

Traditionally, FSH binding to FSHR induces stimulatory Galpha s (Gαs) to activate intracellular adenylate cyclase (AC), cyclic adenosine monophosphate (cAMP) and protein kinase A (PKA) levels. PKA further phosphorylates a large number of cytokines and regulates cAMP-response element binding protein (CREB) transfection in the nucleus to control downstream effector genes, such as UPC1, aromatase and inhibin-A (Walker and Cheng, 2005; Bhattacharya et al., 2021). This classical signaling pathway plays a central role in the biological activities of ovarian granulosa cells and testicular Sertoli cells. At the same time of cAMP/PKA activation, some pro-apoptotic pathways, such as extracellular signal-regulated kinase (ERK)/mitogen activated protein kinases (MAPK) signaling pathways, may be aroused to negatively regulate the generation of cAMP/PKA-dependent steroids (Casarini and Crépieux, 2019). *In vivo* and *in vitro* experiments showed that GnRH stimulation increased FSH levels and upregulated cyclin D1 (CCND1) and cyclin E1 (CCNE1) transcription through the PKA/CREB pathway (Wang et al., 2022). CCND1 and CCNE1 drive the cell cycle and simultaneously accelerate differentiation to promote fat accumulation in adipocytes.

Galpha i (Gαi) is an inhibitory coupling protein of FSHR, which plays a prominent role in FSH regulation of adipocyte metabolism. In adipocytes, β3 adrenergic receptors act on Gαs to activate the cAMP/PKA pathway, increasing ATF2 and UPC1 transcription levels and promoting cell beiging (Wu et al., 2012; Cohen and Spiegelman, 2015). Binding of FSH to Gαi-coupled FSHR blocks the beiging effect of β3 adrenergic pathway (Liu et al., 2015). FSH accelerated the formation of lipid droplets in preadipocytes and human primary adipocytes and increased the expression of a large number of key genes for lipid biosynthesis, including PPARγ, CREB, lipoprotein lipase (LPL), CAAT/enhanced binding proteins (C/EBPa) and fatty acid synthase (FAS). Mechanistically, FSH-treated adipocytes increased the intracellular Ca^2+^ influx. Gαi inhibitor pertussis toxin (PTX) and FSHR knockdown reduced the Ca^2+^ influx and limited lipid biosynthesis. On the other hand, intracellular Ca^2+^ activated phosphatase calcineurin, promoting phosphorylation of protein tyrosine phosphatase non-receptor type 5 (PTPN5). PTPN5 was showed to assist GnRH in inducing FSH secretion through PKA and Gq-phospholipases C (PLC) -P38 MAPK pathway (Wang et al., 2021). Niamh et al. found that FSH acting on Gαi-coupled FSHR increased intracellular lipid droplets in endometrial adenocarcinoma Ishikawa cells. Further research showed that the active interaction between β-arrestin (a key adaptor protein that regulate FSHR signaling) and Gαi during this process was involved in FSH-stimulated lipid biosynthesis (Sayers et al., 2021) (Figure 3). These results indicated that Gαi-coupled FSHR is the key factor that mediates FSH to promote lipid biosynthesis. Inhibition or epigenetic modification of Gαi-coupled FSHR is important for regulating FSH-mediated lipid formation.

### 4.3 FSH regulates cholesterol anabolism

Studies found that changes in body composition in menopausal women were accompanied by dyslipidemia (Jeong and Kim, 2022). High serum total cholesterol (TC) and low-density-lipoprotein cholesterol (LDL-C) were detected in menopausal women and women with premature ovarian failure (Karita et al., 2008; Knauff et al., 2008; Gulhan et al., 2012). Epidemiological study showed that high FSH levels are positively correlated with TC and LDL-C in menopausal women (588 subjects aged 53–73 years), especially in young menopausal women (Serviente et al., 2019). In addition, menopausal women with abdominal obesity had a significantly increased rate of dyslipidemia and associated risk (Pang et al., 2017). This is consistent with the regulation of lipid metabolism and fat distribution by FSH.

Yang et al. (Song et al., 2016) found that menopausal women with high FSH levels (≥78.3 IU/L at baseline) were associated with elevated levels of TC and LDL-C, which could be improved by hormone replacement therapy. In ovariectomized mice, the serum FSH and lipids increased, and the expression of LDL-C receptor (LDLR) in liver tissue decreased. Hepatocyte experiment demonstrated dose-dependent inhibition of intracellular LDLR expression by FSH stimulation. Reduced LDLR expression leads to decreased LDL-C endocytosis and increased circulating LDL levels. Interestingly, while FSH accelerated lipid deposition through PPAR pathway, it activated the cAMP-PKA-pCREB pathway and promoted the transformation of cholesterol to estrogen by upregulating cholesterol master transcription factor sterol regulatory element-binding protein 2 (SREBP2) expression (Cui et al., 2018). Further study revealed that the formation of FSH-FSHR complex activated Gi2α and promoted Akt phosphorylation through phosphatidylinositol-3-hydroxykinase (PI3K) under the regulation of β-arrestin2. Phosphorylated Akt inhibited FOXO1 nuclear transfer and released the SREBP2 transcription site, thereby increasing SREBP2 transcription and expression. Mature SREBP2 increased liver cholesterol synthesis by promoting transcription and expression of cholesterol synthesizes rate-limiting enzyme 3-hydroxy-3-methylglutaryl coenzyme A reductase (HMGCR) (Huang et al., 2021) (Figure 3). Overall, high levels of FSH reduced LDLR expression, resulting in lower LDL utilization and an increase in circulating free LDL. Meanwhile, FSH-FSHR complex increases hepatic cholesterol synthesis through the p-Akt/SREBP2/HMGCR pathway. High levels of cholesterol, especially LDL-C and triglycerides, further activate the inflammatory response of adipocytes (Liu et al., 2021; Santiago-Fernández et al., 2021).

### 4.4 Mesenteric estrogen-dependent adipose genes regulate sex hormone imbalance-induced obesity

FSHR knockdown can directly affect the expression profile of genes related to fat metabolism. FSHR was knocked out to construct obese mice induced by sex hormone imbalance. Transcriptomic analysis of the mesenteric adipose tissue (MAT) found that mesenteric estrogen-dependent adipose (MEDA) genes were involved in fat biosynthesis. Mouse MEDA-4 and MEDA-7 are highly homologous to human MEDA proteins (91% and 71%), encoding 34-kDa and 22-kDa cytosolic secreted proteins, respectively (Zhang et al., 2011; Zhang et al., 2012). The expression of MEDA-4 in mice VAT and omental fat of obese patients was higher than that in subcutaneous fat, and the overexpression of MEDA-4 increased the differentiation of preadipocytes and glucose uptake. Mechanically, MEDA-4 increased lipid accumulation in adipocytes by up-regulating the transcription and expression of PPARγ and many adipogenic factors, such as LPL, hormone-sensitive lipase (HSL), FAS, Acyl-CoA oxidase 1 (Acox1). Although MEDA-7 had a similar tissue distribution of expression to MEDA-4, MEDA-7 attenuated insulin response and glucose uptake in adipocytes by down-regulating glucose transporter-4 (GLUT4). Meanwhile, MEDA-7 was highly expressed in M1 pro-inflammatory macrophages and caused chronic inflammation by upregulating suppressor of cytokine signaling 3 (SOCS3) and monocyte chemotactic protein-1 (MCP-1). Further studies of the relevant genomics of different tissues, especially adipose tissue, will help us to explore the deep link between FSH and fat metabolism.

In conclusion, these studies explained the mechanism of FSH promoting adipogenesis and leading to lipid metabolism disorder in women during MT from different perspectives. Obesity-induced cognitive impairment is well established, but whether FSH is directly involved in cognitive impairment in postmenopausal women or indirectly through lipid metabolism disturbances will be discussed in the next section.

## 5 Epidemiological characteristics of gonadal hormone level and cognitive impairment in menopausal women

Women account for more than 2/3 of AD patients, and the rate of pathology and symptom progression is 2–3 times higher than that of men (Marongiu, 2019). Altered sex hormone levels are thought to be closely associated to cognitive impairment, but some studies showed equivocal results. In older menopausal women (mean age >70), patients with AD were associated with high levels of gonadotropins (Short et al., 2001). The brain tissue showed a significant increase of LH in AD vulnerable neurons (Bowen et al., 2002). A cross-sectional study showed that FSH/estradiol ratio >1.94 is a significant predictor of cognitive impairment in menopausal women (sensitivity 66.5% and specificity, 46.8%) (Hestiantoro et al., 2017). In menopausal women in southern China, higher estrogen levels showed better cognitive performance, but no association with FSH (Hu et al., 2017). However, some studies showed no correlation of sex hormone levels and cognitive performance, and even older women with higher levels of FSH showed better cognitive performance (Luetters et al., 2002; Rodrigues et al., 2008). These results suggest that changes in hormone levels are associated with cognitive impairment in women during the MT, and that differences in results may come from adjusting for age, sex, race, and other factors.

Functional brain imaging revealed a potential correlation between postmenopausal sex hormones and brain function. Functional MRI showed that changes in estradiol and FSH levels in menopausal women affected verbal fluency and frontal cortex activation (Berent-Spillson et al., 2012). The decline of functional connection between bilateral middle occipital gyrus and left inferior parietal gyrus directly affected memory function (Zhang et al., 2018). Estradiol level was negatively correlated with right lingual gyrus regional homogeneity and positively correlated with right superior frontal gyrus, which directly affected cognitive and working memory function (He et al., 2021b). In addition, elevated FSH levels in menopausal women were associated with a reduction in bilateral subcortical volume of the amygdala, resulting in reduced working memory and prolonged response time (Zhang et al., 2021). Together, these studies reveal a potential link between high levels of FSH during the MT and brain structural and functional impairment through neuroimaging.

## 6 Potential mechanisms by which FSH impacts cognition

Changes in sex hormones during the menstrual cycle directly affected hippocampal volume and functional connectivity. Co-localized expression of FSH and its receptors was found in pyramidal neurons in the CA1-CA4 region of the hippocampus and in granular neurons in the dentate gyrus (Chu et al., 2008). Brain MRI scans of women with normal menstrual cycles showed an increase in bilateral hippocampal volume and enhanced functional connectivity with bilateral parietal lobes during the late follicular phase. Compared with early follicular stage, FSH levels decreased rapidly, and brain derived neurotrophic factor (BDNF) levels increased in late follicular stage (Lisofsky et al., 2015). Compared with menstruation with higher FSH levels, women in midluteal phase performed better on language cognitive tasks, and fMRI showed larger activation in the temporal and medial superior frontal. In addition, bilateral hippocampal neuron recruitment was also enhanced in the midluteal phase (Fernández et al., 2003). Rosa et al. found that, from both genetic and non-genetic perspectives, earlier menopausal age was associated with the incidence of AD and the codons 307/680 of FSHR gene with a AS/AS (alanine/serine) genotype had a protective effect on AD susceptibility (Corbo et al., 2011). The FSHR gene variation may affect the interaction between receptor and ligand to reduce the susceptibility of postmenopausal women to AD. FSHR gene modification may be a potential strategy to prevent and treat cognitive impairment during menopause. These findings highlight the impact of FSH fluctuations in the menstrual cycle on brain structure and function, and that such changes may be more pronounced and radical after menopause, manifested in cognitive dysfunction in postmenopausal women.

Kanya et al. found that ovariectomized mice showed a sustained increase in FSH levels and cognitive impairment 3 months later. The expression of Aβ and neurofibrillary tangles-related genes in the hippocampus was up-regulated (Anukulthanakorn et al., 2013). FSHR was found to be involved in the expression of several neurotransmitters in the hippocampus and prefrontal cortex, including aldehyde oxidase 1 (AOX1), retinol dehydrogenase 12 (RDH12), 5-hydroxytryptamine type 3A receptor (HTR3A) and 5-hydroxytryptamine receptor 4 (5-HT4R) (Bi et al., 2020). These cytokines were involved in cognitive and affective disorders. On the other hand, activin A was proved to be a stimulating factor of pituitary to secrete FSH by activating FSHβ and FSHR gene expression (Nakamura et al., 1995; McGillivray et al., 2007; Cossigny et al., 2012). However, activin A as a neurotrophic and neuroprotective factor can promote neurogenesis and synaptic plasticity in AD mice (Lau et al., 2015; Park et al., 2016). This may be an internal homeostatic and self-protective mechanism.

**FIGURE 4:**
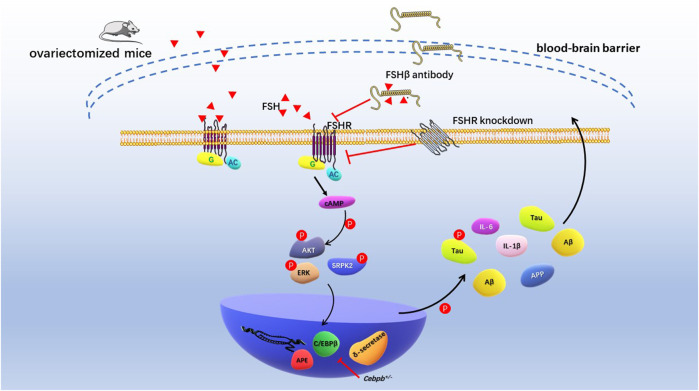
Schematic diagram of FSH promoting cognitive damage of hippocampal and cortical neurons. Ovariectomized mice had significantly elevated levels of follicle-stimulating hormone and impaired cognitive phenotypes. Increased levels of endogenous FSH and exogenous FSH in ovariectomized mice resulted in deposition of Aβ and Tau in the hippocampus and cortex and cognitive impairment. FSH antibodies, FSHR knockdown, and *Cebpb* ^*+/−*^ mice reduced Aβ, Tau, and nerve damage factor expression through AKT/C/EBPβ/δ-secretase pathway, alleviating cognitive impairment in AD model mice.

Recently, Jing Xiong and colleagues conducted the comprehensive and in-depth research of FSH on cognitive function in mice with AD (Xiong et al., 2022). Ovariectomized mice showed ovarian atrophy and significantly increased levels of FSH, Aβ and Tau in brain tissues at 3.5 months of age. After 3–6 months, the learning and spatial memory ability of mice showed distinct deficit, and neurofibrillary tangles appeared 12 months later. Previous studies demonstrated that by targeting 13-amino-acid on the FSHβ sequence (LVYKDPARPNTQK), anti-FSHβ antibody successfully block the effects of FSH on bone cells and adipocytes (Liu et al., 2017; Ji et al., 2018). *In vivo* study showed that the FSHβ antibody reduced Aβ and Tau formation and saved cognitive impairment in ovariectomized mice. Exogenous FSH and FSHβ antibodies not only act peripherally, but also enter the central nervous system through the blood-brain barrier (BBB). Mechanistically, FSH phosphorylates AKT, ERK1/2, and SRPK2 to activate the C/EBPβ-arginine endopeptidase (AEP)/δ-secretase pathway in human and rat neurons. FSHβ antibody treatment inhibited C/EBPβ-AEP/δ-secretase pathway and decreased deposition and phosphorylation of Aβ and Tau. In addition, FSHβ antibody reduced the expression of FSH-induced pro-inflammatory cytokines IL-1β and IL-6. Histologically, blocking FSH reduced neuronal apoptosis and protected dendritic spine and synapse numbers. *Cebpb* ^*+/-*^ mice attenuated the cognitive impairment induced by ovariectomies, again demonstrating that C/EBPβ mediated FSH-induced AD pathology (Figure 4). Taken together, these studies suggested that FSH-FSHR is directly involved in brain nervous system damage *via* the C/EBPβ-AEP/δ-secretase pathway. Antibodies targeting FSH or FSHR can effectively reduce neurofibrillary tangles and inhibit deposition and phosphorylation of Aβ and Tau.

## 7 Potential association between fat metabolism disorder and cognitive impairment in menopausal transition

Obesity is associated with cognitive impairment in adults and increases the risk of AD (Kerwin et al., 2011; Smith et al., 2019). The impaired endothelial function and microvessels rarefaction caused by obesity are closely related to the decreased neurovascular coupling response and the destruction of the BBB (Balasubramanian et al., 2021). In mice with obesity and insulin resistance induced by high fat diet, brain Aβ and neurofibrillary tangles increased, and neuroplasticity decreased (Kothari et al., 2017). Inflammation and stress response caused by obesity are the underlying mechanism of pathological changes in the brain (Wang et al., 2018).

Epidemiological studies showed that obesity and sarcopenic obesity were independently associated with cognitive impairment in elderly Chinese (948 elderly Chinese aged 60–92 years) (Wang et al., 2019). However, another study (1,100 patients aged 60–98 years) showed that being overweight in older adults was significantly associated with a reduced risk of cognitive impairment (OR = 0.458, 95% CI = 0.298–0.703, *p* < 0.001). Importantly, abdominal obesity was found to be significantly associated with an increased risk of cognitive impairment (OR = 1.532, 95% CI = 1.037–2.263, *p* = 0.032), after adjusting for many factors such as age and education level (Hou et al., 2019). A meta-study of 5,060,687 participants from 21 studies found that high waist circumference was associated with a higher risk of cognitive impairment and dementia (HR = 1.10, 95% CI: 1.05–1.15), especially among people over 65 years old (HR = 1.13, 95% CI: 1.08–1.19) (Tang et al., 2021). Thus, central obesity may be a more precise risk factor for cognitive impairment than general obesity. Central obesity is one of the characteristics of fat distribution in menopausal women.

Brain MRI showed that VAT was associated with lower brain volume, even in healthy people (733 participants, mean age, 60 years) (Debette et al., 2010). Combined with the pathogenesis of fat metabolism disorder and cognitive impairment in menopausal women, hormone imbalance may mediate the postmenopausal obesity to aggravate cognitive impairment. Analysis of hormone-related outcomes in menopause suggested that FSH and leptin are associated with brain electrophysiology. Event-related potential (ERP) showed that response latency and response speed were significantly reduced in obese menopausal women with high serum leptin and FSH levels (Braverman et al., 2014). Zsido et al. found that decreased estrogen levels were associated with increased VAT and reduced memory performance during the MT (Zsido et al., 2019).

Animal studies showed that hormone therapy significantly improved the pathological features of dementia in obese middle-aged female rhesus monkeys after surgical menopause (Cervera-Juanes et al., 2022). Estrogen supplementation inhibited pathways associated with neuroinflammation, Aβ deposition, and tau tangles. This suggested that menopausal hormone disruption-induced obesity affects cognitive impairment. Unfortunately, there are no studies on the effect of FSH on cognitive impairment in menopausal fat metabolism disorders.

## 8 Conclusion and future perspectives

Here we summarize the epidemiological findings that high levels of FSH during the MT are associated with abnormal fat metabolism and cognitive impairment. The current studies highlighted that FSHR and Gαi/Ca^2+^/CREB signaling pathways are involved in FSH-mediated lipid generation. As for cognitive impairment, clinical studies found that high levels of FSH are closely related to brain structural and functional damage. Animal studies emphasized the important role of C/EBPβ-AEP/δ-secretase pathway in mediating FSH to promote the pathological progression of cognitive injury. Importantly, epidemiological and partial experimental results suggest that postmenopausal fat metabolism abnormalities have a significant negative impact on cognitive function. Future research is needed to demonstrate how fat formation caused by high levels of FSH during menopause may directly or indirectly impair cognitive function, such as inflammation, mitochondrial damage, oxidative stress, and autophagy changes. In clinical research, since the efficacy and side effects of hormone replacement therapy are not fully understood, lifestyle management, such as exercise, diet, and mood management, should be an important means to prevent lipometabolic disturbance and cognitive impairment in postmenopausal women, as well as some typical menopausal diseases, such as postmenopausal osteoporosis, anxiety, and depression.
